# Supplementary material for: Causal Relationship between Aging and Anorexia Nervosa: A White-Matter-Microstructure-Mediated Mendelian Randomization Analysis
Source: Biomedicines. 2024 Aug 16;12(8):1874. doi: 10.3390/biomedicines12081874 (PMC11351342; doi:10.3390/biomedicines12081874)
Supplement: Supplementary file 1 [file biomedicines-12-01874-s001.zip › Supplementary Figure/Figure S4/Singe SNP plot of MR analysis for TL to ubm-b-1803.pdf]

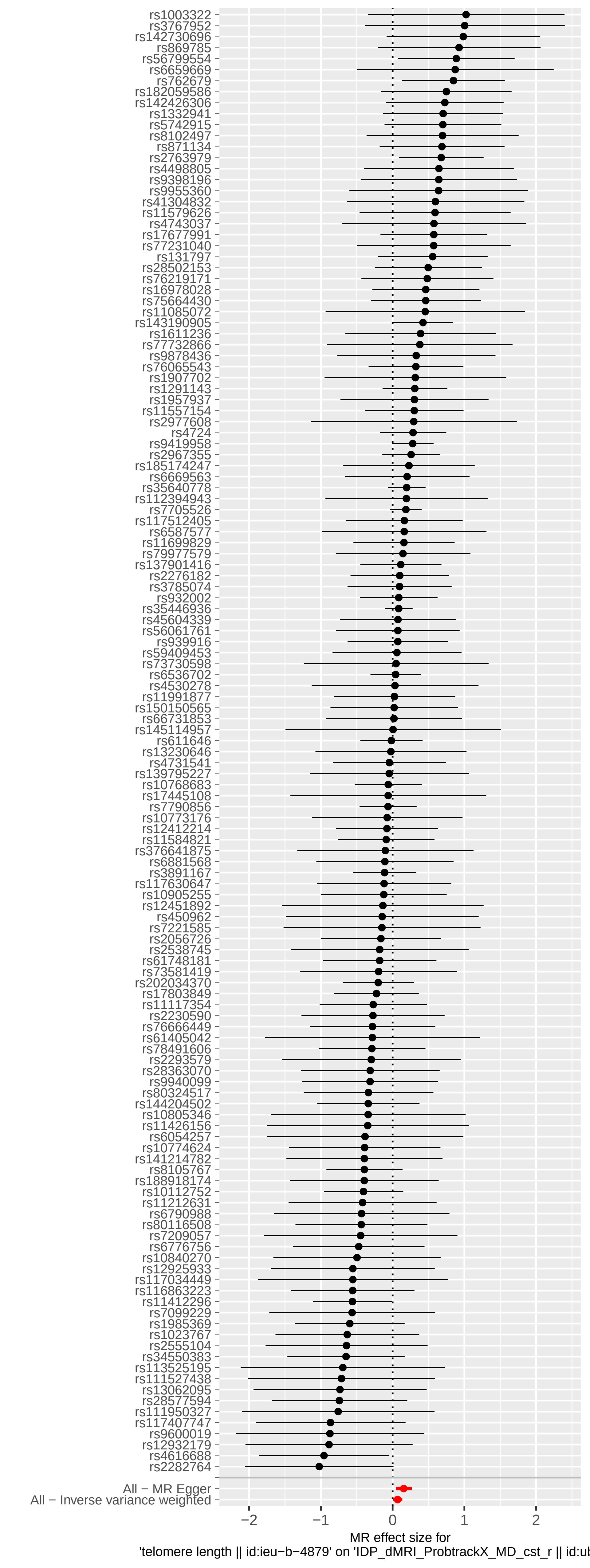
rs1003322
rs3767952
rs142730696
rs869785
rs56799554
rs6659669
rs762679
rs182059586
rs142426306
rs1332941
rs5742915
rs8102497
rs871134
rs2763979
rs4498805
rs9398196
rs9955360
rs41304832
rs11579626
rs4743037
rs17677991
rs77231040
rs131797
rs28502153
rs76219171
rs16978028
rs75664430
rs11085072
rs143190905
rs1611236
rs77732866
rs9878436
rs76065543
rs1907702
rs1291143
rs1957937
rs11557154
rs2977608
rs4724
rs9419958
rs2967355
rs185174247
rs6669563
rs35640778
rs112394943
rs7705526
rs117512405
rs6587577
rs11699829
rs79977579
rs137901416
rs2276182
rs3785074
rs932002
rs35446936
rs45604339
rs56061761
rs939916
rs59409453
rs73730598
rs6536702
rs4530278
rs11991877
rs150150565
rs66731853
rs145114957
rs611646
rs13230646
rs4731541
rs139795227
rs10768683
rs17445108
rs7790856
rs10773176
rs12412214
rs11584821
rs376641875
rs6881568
rs3891167
rs117630647
rs10905255
rs12451892
rs450962
rs7221585
rs2056726
rs2538745
rs61748181
rs73581419
rs202034370
rs17803849
rs11117354
rs2230590
rs76666449
rs61405042
rs78491606
rs2293579
rs28363070
rs9940099
rs80324517
rs144204502
rs10805346
rs11426156
rs6054257
rs10774624
rs141214782
rs8105767
rs188918174
rs10112752
rs11212631
rs6790988
rs80116508
rs7209057
rs6776756
rs10840270
rs12925933
rs117034449
rs116863223
rs11412296
rs7099229
rs1985369
rs1023767
rs2555104
rs34550383
rs113525195
rs111527438
rs13062095
rs28577594
rs111950327
rs117407747
rs9600019
rs12932179
rs4616688
rs2282764
All − MR Egger
All − Inverse variance weighted
−2
−1
0
1
2
MR effect size for
'telomere length || id:ieu-b-4879' on 'IDP_dMRI_ProbtrackX_MD_cst_r || id:ub
